# Supplementary material for: The impact of experimental designs & system sloppiness on the personalisation process: A cardiovascular perspective
Source: PLoS One. 2025 Jun 24;20(6):e0326112. doi: 10.1371/journal.pone.0326112 (PMC12186990; doi:10.1371/journal.pone.0326112)
Supplement: Attachment — Submitted filename: Measurement Response.pdf [file pone.0326112.s002.pdf]

Dear Editor, Reviewer 1 & Dr. Alfonso Santiago,

We would like to thank you all for your detailed and insightful comments on our manuscript. They have undoubtedly increased the impact and clarity of this work. Please find our responses to your comments below in red. You will find the corresponding changes in the article signposted in our responses and in red in the revised article.

We would like to highlight that the article is now in the required template for the journal and the data for the work can now be found at the following Zenodo repository. We thank the editor for these comments and please find the updated funding statements below

Funding Statement: We gratefully acknowledge Polish high-performance computing infrastructure PLGrid (HPC Center: ACK Cyfronet AGH) for providing computer facilities and support within computational grant no. PLG/2024/017108. HS is supported by the UK Engineering and Physical Sciences Research Council (EPSRC) via grant reference EP/Z531297/. PM was funded by the Wellcome Trust [214567/Z/18/Z].

Reviewer #1:.

Major Points:

- Section 2.1.3: Sloppiness certainly affects the choice of numerical strategy (in fact, in numerical analysis, it is related to the concept of “stiffness”). However, the key issue here is different: a sloppy system tends to have elongated or even flat valleys around the minimum, meaning that the minimum is not well-defined. As a result, even small noise in the data could lead to a large region of valid parameter values.

We completely agree with your comment and this is indeed one of the key issues within personalised medicine. The influence of noise on a sloppy system is outside the scope of the work here but is something we are working on offline. We are looking to develop a workflow which examines how the study performed here can be scaled across many different patients with different measurements. We have added a comment to this section to highlight the problems when looking to scale personalised medicine.

- Prior distribution of parameters: The choice of prior distributions is crucial for improving identifiability, especially in low-data regimes. Here, the prior is likely a uniform distribution (though this is not explicitly stated). While a uniform prior is valid in the absence of additional information, the authors should discuss how alternative priors could be incorporated into their workflow. For instance, certain parameter combinations may produce unrealistic physiological outputs (e.g., cardiac output or pressures outside expected ranges). Given population-based distributions of outputs, one could attempt to infer a suitable distribution of input parameters such that random sampling yields the observed output distribution.

This is a fantastic point and we agree it is something which should be investigated in more detail. You are correct here: we utilised a uniform distribution and then produced a Sobol QMC from the prior specified distributions. We have now highlighted this within the text. We have often considered what the best method is to elicit the prior distributions which give realistic outputs. There is significant research on this within the community and the main challenge is the bias that one can place on the input space when trying to find the physiological region for patients. Please see the references below. We have added this insightful comment to the discussion in order to provide more clarity to the reader.

Klymenko, O. V., Kucherenko, S., & Shah, N. (2017). Constrained Global Sensitivity Analysis: Sobol'indices for problems in non-rectangular domains. In *Computer Aided Chemical Engineering* (Vol. 40, pp. 151-156). Elsevier.

Kotidis, P., Demis, P., Goey, C. H., Correa, E., McIntosh, C., Trepekli, S., ... & Kontoravdi, C. (2019). Constrained global sensitivity analysis for bioprocess design space identification. *Computers & Chemical Engineering*, *125*, 558-568.

- Experimental design strategy: My understanding is that each experiment, associated with different measurement sets, is independent of the others. However, suppose we start with the smallest measurement set and fit the parameters: could this information be used to optimize the selection of the next measurement set? Certain parameter combinations may exhibit low sloppiness, which could guide the experimental design.

There is another excellent point and is something that we will consider as future work. In practice, the measurements which are going to be available are largely dominated by the symptoms observed from a patient. Performing such an investigation targeted to a specific condition and seeing how the model performs would be of great interest.

- Section 3.2: Is the clinical data real or synthetically generated? What do you mean by "medically accurate"? Please clarify.

A good point which does need clarification. All the data used here are synthetically generated which allowed us to perform such a large computational investigation. Where our results hold clinical relevance is the groups of measurements and the hierarchy in which we have introduced them. We have devised an iterative scheme which follows the standard clinical pathway for investigation. This has now been clarified in the section to improve readability.

- Tables 5 and 6: These tables are not very accessible. Consider replacing them with a plot. For instance, a "rank plot" could effectively illustrate how parameter importance changes.

We agree there are limitations on these tables due to the amount of information which is present in them. After producing rank plots, we realised a rank plot would lose the actual influence values associated with the parameters in the current tables. Therefore, we would like to keep the tables as the format to display the information details. We have however updated the table captions in order to provide more clarity on how to interpret the tables. We place an example of the rank plot below for table 6 to support our decision.

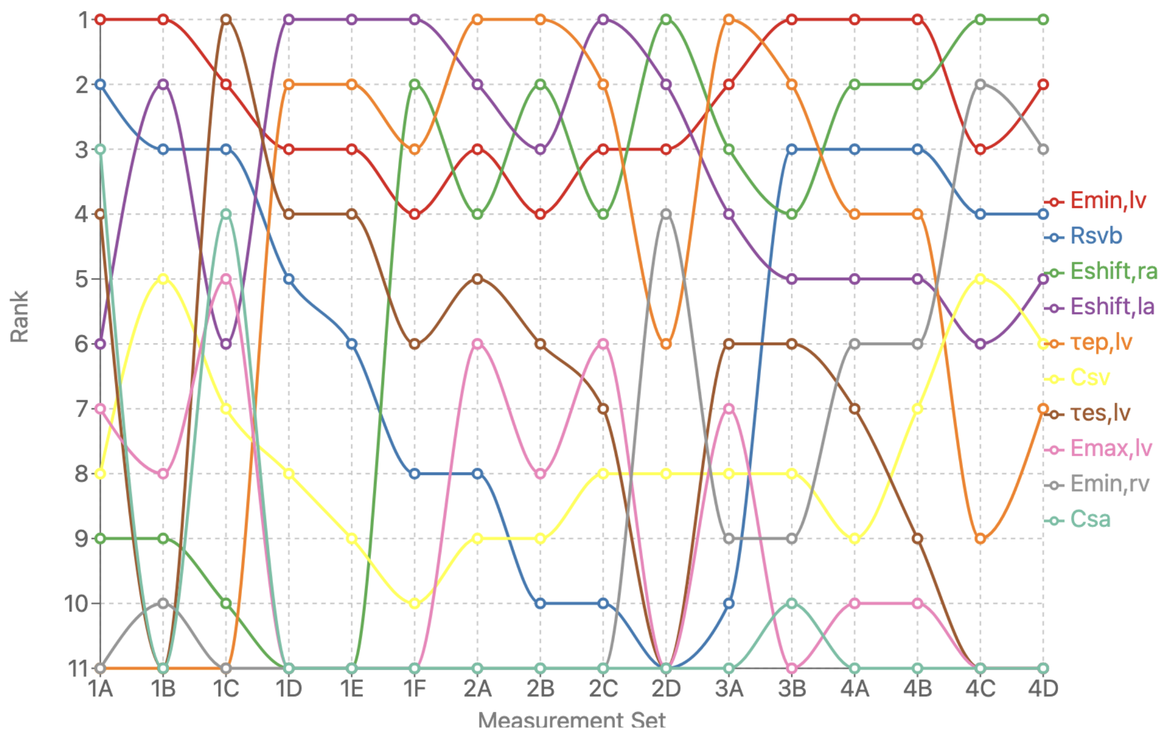

- Page 16: The sentence “Despite the number of discrete measurements outnumbering the number of continuous measurements” is misleading. A continuous measurement is, in principle, infinite-dimensional. While a function can often be approximated using a low-dimensional representation due to correlation, this representation typically has more than one dimension (unless the function is constant).

Mathematically we completely agree with you, however clinically they are often considered as independent objects which contain different information. To enhance clarity, we have modified the sentence as suggested so our article would suit readers from both mathematical and clinical backgrounds.

- Page 20: The paragraph beginning with “One problem of the sloppy analysis” is unclear. Could you refine and clarify this explanation?

We have revised the paragraph to more explicitly address the subjective nature of sloppiness diagnostics and the apparent contradiction between our quantitative findings and intuitive expectations. We have extended our discussion to highlight how the reduction in eigenvalue spread from 10^16 to 10^6 raises important questions about the relationship between mathematical definitions of sloppiness and practical optimisation outcomes. We acknowledge that this discrepancy warrants further investigation, and we have indicated this as an open research question rather than attempting to draw premature conclusions. The revised paragraph now better articulates the nuanced relationship between eigenvalue distributions and parameter optimisation in complex systems.

Minor Points:

- Abstract: Why use “reduced-order” in the first sentence?

We made the choice to utilise this phrasing just to foreshadow the type of model which will be utilised within the work. We agree in reality this is not needed as the work conducted here could be done utilising surrogate modes if a higher dimensional system was investigated. We feel it foreshadows the work nicely and thus is left in the work.

- Abstract: The term sloppiness is not defined. This concept may not be widely known outside statistical modeling communities. Consider providing a brief explanation.
Good point - the abstract has been rewritten in order to incorporate this.

- Author summary: Is this section a requirement for PLOS ONE?
It is not and it has now been removed.

- Terminology: The model is often referred to as a “4-chamber model.” While technically accurate, the term “4-chamber model” nowadays typically refers to a 3D electromechanical model of the heart. Consider using “lumped model” or a similar term to avoid confusion.
This has now been corrected to ‘lumped parameter 4-chamber model’ to ensure no confusion arises.

- Section 2.1.3: The loss function can be visualized in dimensions greater than two using various machine-learning techniques. A simple approach would be to slice the loss landscape with randomly chosen 2D planes.
We view this work as an introductory piece on sloppy analysis for parameter selection and estimation strategies for the cardiovascular research community. Thus, we would like to stick with the 2D example as a straightforward way to aid the explanation.

- Page 12: “poreviosu” -> “previous”
Corrected, thank you.

- Figure sequence: Figure 5 is discussed before Figure 4 (page 14). Consider reordering the discussion or figures for better flow.
Corrected.

- Figure 6: You might add a delineation line between 3D and 4B and clarify that the addition of continuous data is being illustrated.
Added, thanks for the suggestion.

- Page 20: “personlisation” -> “personalisation”
Corrected, thanks.

Reviewer #2:

Summary:

The document “impact of experimental designs & system sloppiness on the personalisation process: a cardiovascular perspective” presents a thorough sensitivity analysis in a simplified model of the cardiovascular system leverages these results to explain the importance on the input parameters depending on the quantities of interest (QoIs) extracted

from (and compared with) the physical system. Particularly, the authors are interested in the sloppiness of the system, this is the separation between the impact of parameters in the outputs, which of course change with the outputs. Particularly, the authors discover that, for this particular model, if the researcher wants to compare QoIs that are continuous clinical measures, there is an increase in the system sloppiness, compared to a discrete measurement approach.

While this work represents a good enough body of work for a publication, I consider there are items that could be improved to increase the technical quality and ease its understanding.

Comments:

- Pag 2, author summary. "We examined how different types of clinical data" is misleading and makes the reader think you are examining clinical data, but the clinical data examination is anywhere in the body of work. You are examining model outputs that are representing clinical data. A model is not an accurate representation of the real world until it's validated for the use condition.
Apologies for this confusion. We agree that this is confusing and misleading for what we are attempting to do. We mean that we are presenting model outputs which correspond to a standard clinical escalation workflow. We have changed the text to reflect this point.

- page 2, paragraph 3. At the time of creating a digital twin, the calibration is only part of the work. Verifying the model is correct or other operational points other than the calibration is critical to confirm the validity of the model. If not, how does the researcher know the results are just an artifact of overfitting? In page 2, paragraph 3, the authors state: "integration of clinical data into an LPM to form a DT, is a task denoted 'the personalisation process' (...) It is this point in the input parameter space which describes a patient's patho/physiological state". But, could the authors ensure the DT will behave as the physical entity in another condition encompassing the patho/physiological state? No, because there is no validation, only one fitting to one calibration point. Improve the paragraph to include this idea.
We have updated the paragraph now to include this point. We think this is a great point and we did not intend for the personalisation process to mean this is the whole basis for DT creation. We have also added into this paragraph about the need for a recursive feedback loop within a DT which as it receives new information the predictions and parameter estimates change.

- Pag 3, paragraph 4: "5. What are the best practices involved in model personalization under uncertainty?" to this reviewer understanding, the authors do not list any set of best practices. Add the best practices in the results or conclusion section.
The best practices for this work can be found at the end of the conclusion, here we have advised on the impact of including discrete or continuous measurements within the model when looking to build a DT.

- Pag 5, paragraph 1, "the final stage of personalization is the optimal estimation of selected input parameters". The authors again assume a fitted model is a correct

model, when the model has not been tested under other conditions.
Updated to include the above points and reinforce again that personalisation is not the final stage within a functioning DT.

● Page 6, section 2.3. “We investigate the impact of varying clinical metrics (...)”. This section is confusing as, so far, the authors have not stated how the real world clinical metrics will be used in conjunction with the model. Specify (probably the optimal place to do this in the document is the introduction) what is the interaction between your model and the physical world, how those real world clinical measures will be used in conjunction with the model virtual clinical metrics.

Thanks for this good point, in order to maintain clarity we have made this connection at the end of the introduction and then reinforced the practical utility of the measurements in section 3.2. Within the work we have tried to follow the standard clinical workflow of iterative tests which correlate with an increase in invasiveness. While we accept in reality no patient is likely to be subjected to this set of tests we feel the novelty involved in performing the first sloppy analysis and subsequent recommendations a sufficient focus for the article.

● Page 8, paragraph 3. “Table 1 provides the parametrization for the model to produce physiological results”. What is the point of defining parameters in a model that will be exclusively used for a global sensitivity analysis (GSA)? Are these the centerpoint of the distributions for the GSA? What are the ranges explored for the GSA? This information is missing. Maybe the authors want to add a graph with the signals (discrete and continuous) for those parameters listed in the table to give meaning to the parameters.

Thank you for pointing this out, the sampling information for how the GSA is computed can now be found at the end of section 3.3.

● Pag 8, paragraph 4 “In tables 2,3 and 4, we display various measurement sets”, after reading the paper it is clear what you are referring to here, but at this point it would be useful to understand that these various sets will be used to execute different sets of experiments.

Thank you for your comment to help us improve the clarity of the work, this has been added to the article now.

● Pag 9, caption in table1 is at the top. It seems to be at the bottom in the rest of the document. Be consistent on the caption position.
Corrected, thank you!

● Page 10, on the noisy blood pressure. This seems such a random thing to do. Why did the authors decide to add the effect of the noisy blood pressure? Why not add noise to every single measurement? Either: (a) remove the noisy BP experiment, (b) do a noisy dataset (for every metric), (c) give a really good argument on why to do the noisy BP experiment and keep it separated from the rest of the body of work (appendix).

We appreciate how the inclusion of this measurement may seem non-natural compared to the rest of the investigation but we feel it provides an additional dimension to the rest of the paper while providing a great foundation for further research. But as the Journal does not allow appendices we have kept this within the main text. The inclusion of noisy blood pressure measurements reveals a critical limitation in standard GSA approaches that assume perfect measurement fidelity. Our findings demonstrate how noise generates significant residual influence across all parameters (evident in Table 7 where all parameters register influence values >0.01), a phenomenon traditional GSA frameworks miss entirely. Though not central to our primary investigation, this component exposes how conventional GSA may overestimate parameter identifiability in clinical scenarios where measurement uncertainty is inevitable. Blood pressure serves as an ideal test case due to its clinical ubiquity and variable acquisition quality. This methodological insight complements our core analysis while establishing foundations for future work on sensitivity analysis frameworks that account for measurement quality—essential for translating cardiovascular digital twins into clinical practice.

- Pag 9, 10,11. Tables 2,3 and 4 have such simple information but are somehow almost incomprehensible. It’s straightforward to read “Set Measurement Added” or “1A Qs”. Please re-design the table or at least draw a horizontal line that makes it easy to infer that the set is 1A and the Qs is included in that set. I understand it may be easy for the authors that wrote the document to infer that, but it was not straightforward for this reviewer to understand the data at first sight.

Apologies for this, we have now modified the table structure and improved the caption of the tables. Hopefully this has improved the readability.

- Pag 13, results. The results of the sensitivity analysis of this paper, are applicable, exclusively to the LPM digital twin julia implementation in this body of work. The authors at any point mention or refer to any code verification or validation (as understood in ASME V&V40), therefore the model could very easily be suffering from model uncertainty (the uncertainty associated with the structure, assumptions, and limitations of the computational model itself). The authors may deem a parameter not sensitive just because there is a coding or hypothesis error. This has to be clearly stated in the introduction or limitations of the document.

This is an excellent point and one which absolutely should be included. We have added this comment within the discussion section highlighting the full work which is required for computational models to be validated as a functioning DT.

- Pag 19, Results. What is the real world utility of these results other than being a computational experiment? The work needs a real application example after the mixed measures example. While the document explains what inputs are relevant depending on the outputs to compare, there is not even a remote hint on how such a DT could be used in practice to overcome issues like estimating the arterial Resistance.

We consider this research as more fundamental research and characterising the behaviour of such a model which is why we have performed the first sloppy analysis of such a model. The understanding gained from this investigation will underpin our future research in developing a functioning DT which provides recursive feedback. We have mentioned what

the next steps are within the discussion and what future barriers one may have to overcome in order for such DT to be deployed. We have tried to reinforce our intentions of the work at the end of the introduction.

- Page 20, “the increasing number of equally significant parameters is unsurprising”, please argue why the cross correlation on a larger dataset is not an explanation for this increasing number of equally significant parameter sets.

While cross-correlation between measurements in larger datasets might seem like a plausible explanation for the increasing number of equally significant parameters, several lines of evidence suggest this is not the primary mechanism at work. First, our experimental design deliberately incorporated physiologically distinct measurements that capture different aspects of cardiovascular function—from pressure dynamics to volume changes across multiple cardiac chambers and vascular beds. The observed changes in parameter significance distributions persist even when comparing measurement sets with minimal expected correlation (such as comparing discrete ejection fraction metrics with continuous pressure waveforms). Second, the increased parameter significance correlates strongly with the specific physiological subsystems being measured, rather than with the total measurement count alone, suggesting true additional constraint rather than redundant information. For instance, when adding pulmonary system measurements (set 4C), we observe significant shifts in previously unidentifiable pulmonary parameters, indicating genuine new constraints from these measurements. Third, if cross-correlation were the dominant effect, we would expect parameter significance to saturate quickly as measurement count increases, yet we observe continued evolution of the significance distribution across very large measurement sets. Nevertheless, some degree of measurement cross-correlation likely does occur, particularly within continuous waveform measurements of closely coupled cardiovascular subsystems

Acknowledgement of this point has been added to the discussion to reflect this possibility and point towards future work in looking to identify cross correlations to further enhance experimental design investigations.

- Page 21, “136.8 million times”. The authors know there is no need to explicitly execute the model when they could have used polynomial chaos expansion or other similar techniques. Explain why you decided to take the explicit calculation approach instead of using other techniques.

This is a fair and accurate point. For us, computing the sensitivities directly avoids the uncertainty which can be introduced through emulation. While we accept the results are likely to be identical at a fraction of the computational cost we want to do the calculations directly due to what is planned in our future work. In future we would like to identify unstable/unphysiological regions of input parameter space, which when using an emulator assumes a fully continuous surface. We look forward to publishing this comparison between emulated and direct sensitivities soon! We have expanded on this point in the discussion to reflect this point.
