# Supplementary material for: The impact of experimental designs & system sloppiness on the personalisation process: A cardiovascular perspective
Source: PLoS One. 2025 Jun 24;20(6):e0326112. doi: 10.1371/journal.pone.0326112 (PMC12186990; doi:10.1371/journal.pone.0326112)
Supplement: Attachment — Submitted filename: Review.docx [file pone.0326112.s001.docx]

**On the review of “impact of experimental designs & system sloppiness on the personalisation process: a cardiovascular perspective”**

**Summary:**

The document “impact of experimental designs & system sloppiness on the personalisation process: a cardiovascular perspective” presents a thorough sensitivity analysis in a simplified model of the cardiovascular system leverages these results to explain the importance on the input parameters depending on the quantities of interest (QoIs) extracted from (and compared with) the physical system. Particularly, the authors are interested in the sloppiness of the system, this is the separation between the impact of parameters in the outputs, which of course change with the outputs. Particularly, the authors discover that, for this particular model, if the researcher wants to compare QoIs that are continuous clinical measures, there is an increase in the system sloppiness, compared to a discrete measurement approach.

While this work represents a good enough body of work for a publication, I consider there are items that could be improved to increase the technical quality and ease its understanding.

**Comments:**

- Pag 2, author summary. “*We examined how different types of clinical data*” is misleading and makes the reader think you are examining clinical data, but the clinical data examination is anywhere in the body of work. You are examining model outputs that are representing clinical data. A model is not an accurate representation of the real world until it’s validated for the use condition.
- page 2, paragraph 3. At the time of creating a digital twin, the calibration is only part of the work. Verifying the model is correct or other operational points other than the calibration is critical to confirm the validity of the model. If not, how does the researcher know the results are just an artifact of overfitting? In page 2, paragraph 3, the authors state: “*integration of clinical data into an LPM to form a DT, is a task denoted ‘the personalisation process’ (...) It is this point in the input parameter space which describes a patient’s patho/physiological state*“. But, could the authors ensure the DT will behave as the physical entity in another condition encompassing the patho/physiological state? No, because there is no validation, only one fitting to one calibration point. Improve the paragraph to include this idea.
- Pag 3, paragraph 4: “*5. What are the best practices involved in model personalization under uncertainty?*” to this reviewer understanding, the authors do not list any set of best practices. Add the best practices in the results or conclusion section.
- Pag 5, paragraph 1, “the final stage of personalization is the optimal estimation of selected input parameters”. The authors again assume a fitted model is a correct model, when the model has not been tested under other conditions.
- Page 6, section 2.3. “We investigate the impact of varying clinical metrics (...)”. This section is confusing as, so far, the authors have not stated how the real world clinical metrics will be used in conjunction with the model. Specify (probably the optimal place to do this in the document is the introduction) what is the interaction between your model and the physical world, how those real world clinical measures will be used in conjunction with the model virtual clinical metrics.
- Page 8, paragraph 3. “*Table 1 provides the parametrization for the model to produce physiological results*”. What is the point of defining parameters in a model that will be exclusively used for a global sensitivity analysis (GSA)? Are these the centerpoint of the distributions for the GSA? What are the ranges explored for the GSA? This information is missing. Maybe the authors want to add a graph with the signals (discrete and continuous) for those parameters listed in the table to give meaning to the parameters.
- Pag 8, paragraph 4 “*In tables 2,3 and 4, we display various measurement sets*”, after reading the paper it is clear what you are referring to here, but at this point it would be useful to understand that these various sets will be used to execute different sets of experiments.
- Pag 9, caption in table1 is at the top. It seems to be at the bottom in the rest of the document. Be consistent on the caption position.
- Page 10, on the noisy blood pressure. This seems such a random thing to do. Why did the authors decide to add the effect of the noisy blood pressure? Why not add noise to every single measurement? Either: (a) remove the noisy BP experiment, (b) do a noisy dataset (for every metric), (c) give a really good argument on why to do the noisy BP experiment and keep it separated from the rest of the body of work (appendix).
- Pag 9, 10,11. Tables 2,3 and 4 have such simple information but are somehow almost incomprehensible. It’s straightforward to read “Set Measurement Added” or “1A Qs”. Please re-design the table or at least draw a horizontal line that makes it easy to infer that the set is 1A and the Qs is included in that set. I understand it may be easy for the authors that wrote the document to infer that, but it was not straightforward for this reviewer to understand the data at first sight.
- Pag 13, results. The results of the sensitivity analysis of this paper, are applicable, exclusively to the LPM digital twin julia implementation in this body of work. The authors at any point mention or refer to any code verification or validation (as understood in ASME V&V40), therefore the model could very easily be suffering from model uncertainty (the uncertainty associated with the structure, assumptions, and limitations of the computational model itself). The authors may deem a parameter not sensitive just because there is a coding or hypothesis error. This has to be clearly stated in the introduction or limitations of the document.
- Pag 19, Results. What is the real world utility of these results other than being a computational experiment? The work needs a real application example after the mixed measures example. While the document explains what inputs are relevant depending on the outputs to compare, there is not even a remote hint on how such a DT could be used in practice to overcome issues like estimating the arterial resistance.
- Page 20, “*the increasing number of equally significant parameters is unsurprising*”, please argue why the cross correlation on a larger dataset is not an explanation for this increasing number of equally significant parameter sets.
- Page 21, “*136.8 million times*”. The authors know there is no need to explicitly execute the model when they could have used polynomial chaos expansion or other similar techniques. Explain why you decided to take the explicit calculation approach instead of using other techniques.
